# Assessment of the Cytotoxic Activity of Triphala: A Semisolid Traditional Formulation on HepG_2_ Cancer Cell Line

**DOI:** 10.1155/2021/6689568

**Published:** 2021-08-11

**Authors:** Ali Sahragard, Zohreh Alavi, Zohreh Abolhassanzadeh, Mahmoodreza Moein, Afshin Mohammadi-Bardbori, Mahmoud Omidi, Mohammad M. Zarshenas

**Affiliations:** ^1^Medicinal Plants Processing Research Center, Shiraz University of Medical Sciences, Shiraz, Iran; ^2^Department of Phytopharmaceuticals (Traditional Pharmacy), School of Pharmacy, Shiraz University of Medical Sciences, Shiraz, Iran; ^3^Medical Plants Research Center, Basic Health Sciences Institute, Shahrekord University of Medical Sciences, Shahrekord, Iran; ^4^Department of Pharmacognosy, School of Pharmacy, Shiraz University of Medical Sciences, Shiraz, Iran; ^5^Department of Pharmacology and Toxicology, School of Pharmacy, Shiraz University of Medical Sciences, Shiraz, Iran; ^6^Department of Pharmacology and Toxicology, Faculty of Pharmacy, Hormozgan University of Medical Sciences, Bandar Abbas, Iran; ^7^Epilepsy Research Center, Shiraz University of Medical Sciences, Shiraz, Iran

## Abstract

Cancer chemotherapies may result in resistance, and therefore, contemporary treatments including natural products may find an increasing consideration. As per Persian medicine (PM), many natural products have been used for malignant and chronic diseases. Triphala, with a combination of *Terminalia chebula* Retz., *Terminalia bellirica* Retz., *Phyllanthus emblica* L., and honey, is a multi-ingredient traditional formulation attributed to anticancer activities in PM. This study is aimed at evaluating the cytotoxic activity of this preparation on HepG_2_, the human liver cancer cell line. Hydroalcoholic extracts were prepared from the formulation and its components. Compared with the control and Cisplatin, the extracts were tested using MTT assay at different concentrations. All concentrations of the preparation, as well as Cisplatin, were effective significantly against HepG_2_ cells. All extract preparations at multiple concentrations were significantly effective as evidenced by MTT assay when compared to the control group. The IC_50_ level for Triphala extract was 77.63 ± 4.3 *μ*g/ml. Based on the results, Triphala and its components have cytotoxic activity on the HepG_2_ cancer cell line and they can reduce the survival rate significantly.

## 1. Introduction

In developed and developing countries, one of the main causes of death is cancer. Cancer is the abnormal growth of human cells and is also a factor to interfere with human healthy and functional cell lines. The occurrence of cancer is mainly due to imbalances in many organs and requires huge control costs yearly [1]. In addition to expensive costs and lack of widespread and easily available cancer drugs, many side effects have also been reported for these medicines [2].

In recent decades, the role of medicinal plants as an alternative or adjuvant therapy to conventional drugs in the control and prevention of cancer and related complications has received widespread attention [3]. Documents and reports of complementary medicine around the world are valuable for new drug discovery and management of various diseases. Persian medicine (PM), as one of the well-known and developed sections of complementary medicine, has introduced medicines derived from medicinal plants for the treatment of various diseases [4].

In PM, many preparations have been reported to be effective against organ dysfunction, indirectly represented to have occurred concerning cancer and malignancies. *Itrifal*-*saghir* is one of those formulations consisting of *Terminal chebula* Retz. (two fruit types as yellow and black), *Terminalia bellirica* Retz., and *Phyllanthus emblica* L. This preparation is widely used in PM and has been mentioned to increase physical strength as well as rehabilitate the body against cancer and malignancies [5]. Components of *Itrifal*-*saghir* have various pharmacological effects. *P. emblica* fruit has antibacterial and antioxidant [6], blood pressure lowering [7], and gastrointestinal wound healing activities [8], as well as protective effects on certain chemotherapy drugs [9]. The effects of *T. bellirica* include reducing fever and pain [10], antidepressant effects [11], lowering blood sugar [12], and inhibiting cancer cell growth [13].

*T. chebula*, which is a very popular medicinal fruit, also has various effects including analgesic and anti-inflammatory effects [14], cancer cell growth inhibition [15], and antibacterial activities [16].

On the other hand, the combination of these three fruits called *Tri-Pahle* or Triphala also has several pharmacological effects. Anti-inflammatory effects on arthritis in rats [17], antimicrobial activities [18], protective effect on liver cells [19], antioxidant [20], and antiobesity properties [5] have been proved by current medicine. Antitumor effects have also been observed from a mixture of these three fruits in Triphala [21].

Due to its beneficial effects and practice in PM, the current study was conducted to evaluate the cytotoxic activity of Triphala.

## 2. Materials and Methods

### 2.1. Studied Plants

Samples of each constituent were purchased from a medicinal plant market in Shiraz (Southern Iran) and transferred to the Department of Phytopharmaceuticals at Shiraz School of Pharmacy. As shown in Table 1, a voucher number was subsequently specified after the authentication of the constituents.

### 2.2. Formulation and Preparation of Product Components

The formulation of Triphala is composed of the above ingredients in a ratio of 1 : 1 : 1. After weighing the milled samples, they were mixed in the desired ratio so that the final weight of the resulting powder was 1 kg, and finally, Triphala was prepared in the form of a semisolid oral formulation (confection) by adding honey equal to the weight of the entire powder. The product was stored in a sealed glass container at room temperature and protected from light for 40 days to be prepared for consumption [22].

### 2.3. Extraction and Concentration

The ultrasonic bath was used to yield the extract of Triphala preparation and each constituent. An ethanol-water solution (80 : 20 *v*/*v*) was used to prepare the extract (at 40°C for 30 minutes) either for final formulation or for each ingredient. At this stage, the solvent was renewed 3 times for each extract to achieve maximum efficiency. The resulting hydroalcoholic extracts were first transferred to a rotary device and concentrated using a Buchner funnel and vacuum filter, which did not contain any possible particles from the extraction process. The final extract for each sample was then concentrated under vacuum by a rotary evaporation device and finally was dried in a speed-vacuum apparatus. The percentage yield was calculated as follows:

(1) $$\begin{array}{c} \text{Percentage yield}=\left(\frac{\text{Weight of extract}}{\text{Weight of plant parts}}\right)\times 100. \end{array}$$

### 2.4. Cell Culture

In this study, the HepG_2_ cancer cell line was purchased from Iran's National Cell Bank, Pasteur Institute in Tehran. They were passaged 26–31 times in DMEM medium, containing 10% fetal bovine serum (FBS), 100 mM sodium pyruvate, 1.5 g/l sodium bicarbonate, and 1% penicillin-streptomycin antibiotic, incubated at 37°C, 5% carbon dioxide, and sufficient humidity.

For various tests, when the cells reached at least 70% cell growth, they were separated from the bottom of the flask by trypsin-ethylenediaminetetraacetic acid (EDTA) and centrifuged at 1300 rpm for 5 minutes [23–25].

Cell viability was determined by trypan blue. A cell suspension containing “*X* × 10,000,000” cells was mixed with trypan blue and stained, and nonstained cells were counted [26]. After ensuring that the cells were not infected, cells with viability above 90% were used for testing.

### 2.5. Clonogenicity Assay

In this method, 500 cells were incubated in a 15 cm^3^ sterile Petri dish for 7 days, and after the incubation period was over and the formation of cell colonies was ensured by a microscope, treatment with different extracts at the mentioned concentrations was started. After 72 hours of cell treatment and ensuring that the number of cell colonies could be distinguished, the Petri dishes were transferred to the laminar flow hood under aseptic conditions, and then, the supernatant medium was gently removed and washed twice with neutral PBS.

Then, the cells were treated with 5 ml of 10% formalin buffer for 30 minutes to be fixed. Afterward, the formalin buffer was gently removed, and 5 ml 1%*W*/*V* crystal violet dye was added to the cell colonies. They were incubated at room temperature for 60 minutes. Thereafter, the dye medium was gently removed, and the Petri dish containing the stained cell colonies was carefully immersed in a container filled with double-distilled water to wash off the excess crystal violet dye. The number of colonies was calculated using a stereomicroscope. The efficiency of colony formation and surviving fraction were evaluated using the following formulas:

(2) $$\begin{array}{c} \text{Colony Forming Efficiency}\left(\text{CFE}\right)=\frac{\text{number of colonies counted}}{\text{number of cells plated}},\\ \text{Surviving fraction}=\frac{\left(\text{number of colonies counted}/\text{number of cells plated}\right)}{\text{CFE}\left(\text{control}\right)}. \end{array}$$

The difference in the number of colonies after exposure to the drugs or the absence of drugs is a sign of drug cytotoxicity. Finally, the results were analyzed [27].

### 2.6. MTT [3-(4,5-Dimethylthiazol-2-yl)-2,5-diphenyltetrazolium Bromide] Assay

To perform the MTT assay, the following steps were carried out [28].

1. In each well (96-well plate), 100 microliters of DMEM/F12 medium containing 10^5^ cells from the cell line was added (for each concentration of test substance, including Cisplatin and extract, 3 wells were considered; Cisplatin has been used as a standard)
2. The 96-well plate containing the implanted cell line was incubated for 24, 48, and 72 hours to allow the cells to reach the logarithmic growth phase and adhere to the bottom of the plate
3. During the incubation period, the cells were checked to ensure how the cells grow and whether they are not infected
4. At the end of the incubation period, 50 *μ*l of Cisplatin and the extracts were added to the 3 wells
5. After adding the desired concentration of the test drug to the cells implanted in each well, we put a 96-well plate in the incubator for the second 24, 48, and 72 hours to make the cells fully contact the test drugs (for each drug and each concentration, three incubation times were considered)
6. The 96-well plates were taken out from the incubator, a pipette was used to remove the supernatant medium carefully from the well, and finally, the cells were washed with 0.5 ml sterile saline three times
7. One hundred microliters of MTT solution was added to each well; the 96-well plates were covered with aluminum foils and placed in the incubator for 4 hours
8. After the required time, MTT solution was removed from the wells and 50 *μ*l DMSO solution diluted in DMEM/F12 medium was added (250 *μ*l DMSO in 5 ml DMEM/F12 medium) to each well for 15 minutes
9. After 15 minutes, the uptake of stained cell colonies in each well was checked by an ELISA reader at two wavelengths of 570 and 690 nm
10. All data are expressed as a percentage of cytotoxicity against HepG_2_ cells. They were calculated as mean ± standard deviation, and the experiment was repeated 3 times for each sample

### 2.7. IC_50_ Calculation

The SPSS Ver. 20 statistical software was used to calculate the viability percentage of each normal and cancer cells after treatment with Cisplatin and the extracts, as well as the cytotoxicity and growth inhibitory efficacy (IC_50_) of these drugs after the MTT test.

### 2.8. Statistical Method Used

All statistical calculations used to compare IC_50_s were performed by Prism Ver. 3 statistical software, using the nonlinear regression method. SPSS Ver. 20 software was used to compare the data with the one-way analysis of variance (ANOVA) method and its related posttest (Tukey-Kramer multiple understanding test). The same graphics program was used to draw charts.

### 2.9. Statistical Analysis

The sample size was calculated as nearly 6 where *Z* = 1.96, accuracy (*d*) = 1, and standard deviation = 1.25. After collecting data using the SPSS Ver. 20 computer program, the one-way or two-way ANOVA statistical test, Student *t*-test, and Tukey-Kramer multiple comparisons were used to compare the test groups. Values of *p* < 0.05 were considered to be statistically significant.

## 3. Results

### 3.1. The Percentage Yield of Extracts

The percentage yield of *T. chebula* (yellow and black fruits), *T. bellirica*, *P. emblica*, and Triphala (the preparation) extracts is shown in Table 2.

### 3.2. The Effect of Different Concentrations of Extracts on the Colonization Index

To study the impact of the extracts on the colonization index, the colonies were treated with different concentrations of *T. chebula* (yellow and black fruits), *T. bellirica*, *P. emblica*, and Triphala extracts. The effect of the extracts on the colonization index is shown in Table 3.

#### 3.2.1. MTT Results

**Figure 1:**
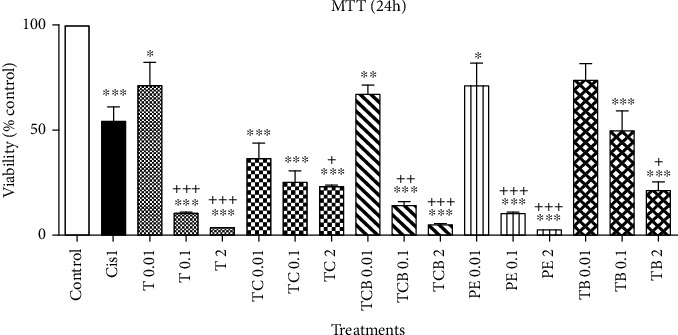
Effect of Triphala hydroalcoholic extract and its compounds on HepG_2_ cell proliferation using MTT assay. ∗, ∗∗, and ∗∗∗ indicate a significant difference compared to the control group (respectively, *p* < 0.05, *p* < 0.01, and *p* < 0.001). +, ++, and +++ indicate a significant difference compared to Cisplatin (respectively, *p* < 0.05, *p* < 0.01, and *p* < 0.001). PE: *P. emblica*; TC: *T. chebula* (yellow fruit); TCB: *T. chebula* (black fruit); TB: *T. bellirica*; T: Triphala; Cis: Cisplatin.

Figure 1 shows the in vitro cytotoxic activity of Triphala extracts and its components. All extracts showed cytotoxic activity against HepG_2_ cells. However, the extract of *P. emblica* and Triphala at the concentrations of 2 mg/ml showed a higher cytotoxic effect.

### 3.3. IC_50_ Level for the Extracts and Cisplatin

Table 4 represents the IC_50_ level of Cisplatin and Triphala extract and its components on HepG_2_ cells. The IC_50_ level of Cisplatin and the extracts against HepG_2_ cells was as follows, viz., *T. bellirica* > *T. chebula* (yellow fruit) > *T. chebula* (black fruit) > Triphala > *P. emblica* > Cisplatin.

## 4. Discussion

Due to the high mortality rate of cancer and many side effects of chemotherapy and radiotherapy drugs, many patients seek alternative or complementary therapies [29, 30]. For many cancers, the most important preventive measures are diet changes, smoking cessation, treatment of inflammatory diseases, and immune-boosting supplements [30]. Nowadays, due to discoveries in the field of cell biology, researchers are trying to find new suitable chemotherapy methods and treat cancer with minimal or no side effects. Chemotherapy is the main treatment method to control the advanced stage of malignant tumors, and it is also a preventive agent in possible metastases, which can cause serious toxicity to the normal cells of the body [31, 32].

Plants have always been used to treat many diseases of humans and animals. Plants maintain individual health and physical strength as well as curing diseases. For example, they can kill cancer without causing toxicity. More than 50% of modern drugs used clinically are derived from natural products that can control cancer [33].

The WHO estimates that more than 80% of people in developing countries rely on traditional medicine to provide primary care [34]. In the last few decades, herbal and traditional medicine has been accepted all over the world and has influenced the medical community and international trade at the same time. Herbal medicines play a very important role in the health system of many people in the world [35].

Persian medicine (PM) sources have introduced many drugs to enhance human health, thereby controlling and healing chronic diseases. According to the reports from PM, many plants such as *T. chebula* and *P. emblica* have tonic effects and can boost the body's immunity [36–40]. On the other hand, new research confirms their properties including antibacterial effects [41, 42], boosting the immune system [43, 44], anticancer effects [43–45], and antioxidants effects [41, 42, 46, 47].

In traditional Indian medicine resources, a compound called Triphala is known, including *T. chebula*, *T. bellirica*, and *P. emblica* [47]. Previous studies on Triphala have shown that it has anticancer effects on MCF-7 [48–50], PC-3, S115, DU-145 [49], T47D [50], and Barcl-95 cell lines [48]. Tannins, as effective main ingredients in the composition of these plants, may play a critical role in this approach. Tannin compounds of *T. chebula* include chebulic acid, chebulinic acid, chebulagic acid, gallic acid, and ellagic acid [43]. Those in *T. bellirica* include ellagic acid, gallic acid, ethyl gallate, galloyl glucose, and chebulagic acid [51, 52], and those in *P. emblica* include ellagic acid and gallic acid [53].

Tannins, whether in total tannins or pure compounds, play an important role in the prevention and treatment of cancer. Tannins have very bright prospects in the development of new anticancer drugs as preventive and therapeutic agents [54, 55].

MTT is a colorimetric test used to evaluate the metabolic activity of cells. To study the cytotoxic effects of drugs on the growth and proliferation of cancer cells and determine the IC_50_ of these compounds, the MTT colorimetric method is used. MTT powder is a water-soluble tetrazolium salt. When dissolved in a medium culture without red phenol or PBS buffer, a yellow compound is formed. The basis of this test is that the enzyme mitochondrial succinate dehydrogenase of living cells breaks down MTT salt. The result is the formation of insoluble crystals of purple formazan, which are dissolved by dimethyl sulfoxide (DMSO). The more cells and the more active they are, the more colors will be created. An ELISA reader is used to measure the absorption of the purple light appearing at 490-540 nm [56]. According to Table 4 and regarding the CFE associated with each sample and the survival rate, it can be concluded that all concentrations of Triphala and its components, as well as Cisplatin, were effective on cancer cells and they have significantly reduced the survival rate.

It is often mentioned that MTT assay is more facilitated than other methods for studying cell proliferation and can be implemented using facilities available in most laboratories. Besides, all test steps are performed on 96-well cell cultures, and the results are read by an ELISA reader, so a large number of samples can be tested at the same time [28]. According to Figure 1, in response to MTT, all concentrations of Triphala extract and its components were significantly different from the control group.

It is considered that all ingredients except *T. bellirica* A (IC_50_ = 129.3 ± 7.8 *μ*g/ml) showed effective values against the cancer cell line. However, the large difference between the IC_50_ values for extracts and positive control (Cisplatin) is related to the total amount of phytoconstituents in each component extract and that of the final formulation (Triphala) that may dilute the effects. Concerning Cisplatin, we encounter a pure potent medicament.

Considering that this study was carried out on the cytotoxic activity of a multi-ingredient herbal formulation on a specific cancer cell line, while the results were promising in comparison with Cisplatin as the positive control, the extract of this formulation can be introduced as a natural cytotoxic agent and an adjuvant to other related drugs to reduce their concentration and help to boost the immune system due to its nutritional effects. However, it should be noted that the activity and impact of this extract on normal cells must be examined.

Generally, based on the results of this study, it can be concluded that all concentrations of Triphala and its components have cytotoxic activity on the HepG_2_ cancer cell line and they can significantly reduce the survival rate as evidenced by MTT assay. Further research needs to be carried out to identify the active ingredients that cause the cytotoxic activity of this traditional product.

## Figures and Tables

**Table 1:** Purchased plants and their voucher number.

No.	Scientific name	Voucher number
1	*Terminal chebula* Retz. (yellow fruit)	pm 299
2	*Terminal chebula* Retz. (black fruit)	pm 922
3	*Terminalia bellirica* Retz.	pm 895
4	*Phyllanthus emblica* L.	pm 982

**Table 2:** Percentage yield of Triphala extract and its components.

Samples	Weight of plant parts (g)	Weight of extracts (g)	Percentage yield (%)
*P. emblica*	25	4.7	18.8
*T. bellirica*	25	4.1	18.4
*T. chebula* (yellow fruit)	25	4.3	17.2
*T. chebula* (black fruit)	25	4.2	16.8
Triphala	25	4.5	18

**Table 3:** The effects of different extract concentrations on the colonization index.

Treatment	Cell plated	Number of colonies			CFE (%)	Survival fraction
		1	2	3		
Control	500	139	147	142	0.286 ± 0.008	1
Cis (1 *μ*g/ml)	500	21	19	24	0.043 ± 0.005^∗∗∗^	0.15
PE (0.1 mg/ml)	500	20	27	32	0.053 ± 0.012^∗∗∗^	0.18
PE (0.01 mg/ml)	500	57	68	69	0.13 ± 0.013^∗∗∗^	0.45
TB (0.1 mg/ml)	500	34	21	27	0.055 ± 0.013^∗∗∗^	0.19
TB (0.01 mg/ml)	500	93	112	116	0.214 ± 0.024^∗∗∗^	0.75
TCB (0.1 mg/ml)	500	36	31	29	0.064 ± 0.007^∗∗∗^	0.22
TCB (0.01 mg/ml)	500	76	87	91	0.17 ± 0.01^∗∗∗^	0.59
TC (0.1 mg/ml)	500	31	20	19	0.047 ± 0.01^∗∗∗^	0.16
TC (0.01 mg/ml)	500	27	38	43	0.072 ± 0.01^∗∗∗^	0.25
T (0.1 mg/ml)	500	39	37	41	0.078 ± 0.004^∗∗∗^	0.27
T (0.01 mg/ml)	500	66	57	51	0.116 ± 0.015^∗∗∗^	0.41

PE: *P. emblica*; TC: *T. chebula* (yellow fruit); TCB: *T. chebula* (black fruit); TB*: T. bellirica*; T: Triphala; Cis: Cisplatin. ^∗∗∗^Significant difference (*p* < 0.001) compared to the control group.

**Table 4:** IC_50_ level of ethanolic extract of Triphala and its components and Cisplatin on HepG_2_ cells.

Tested material	IC_50_^a^ (*μ*g/ml)
Cisplatin^b^	3.32 ± 0.06
*P. emblica*	76.99 ± 5.1
*T. bellirica* ^b^	129.3 ± 7.8
*T. chebula* (yellow fruit)	81.28 ± 4.7
*T. chebula* (black fruit)	81.15 ± 3.9
Triphala	77.63 ± 4.3

All determinations were done in triplicate, the standard deviation for 95% confidence. ^a^IC_50_: the concentration of drug required for 50% inhibition. ^b^Reference standard.

## Data Availability

Respectful readers may contact the corresponding author in order to receive all shown or not shown data (zarm@sums.ac.ir).
